# The *Bryopsis hypnoides* Plastid Genome: Multimeric Forms and Complete Nucleotide Sequence

**DOI:** 10.1371/journal.pone.0014663

**Published:** 2011-02-14

**Authors:** Fang Lü, Wei Xü, Chao Tian, Guangce Wang, Jiangfeng Niu, Guanghua Pan, Songnian Hu

**Affiliations:** 1 Institute of Oceanology, The Chinese Academy of Sciences (IOCAS), Qingdao, China; 2 Beijing Genomics Institute, The Chinese Academy of Sciences (BGICAS), Beijing, China; 3 Graduate University of the Chinese Academy of Sciences, Beijing, China; 4 College of Marine Science and Engineering, Tianjin University of Science and Technology, Tianjin, China; Michigan State University, United States of America

## Abstract

**Background:**

*Bryopsis hypnoides* Lamouroux is a siphonous green alga, and its extruded protoplasm can aggregate spontaneously in seawater and develop into mature individuals. The chloroplast of *B. hypnoides* is the biggest organelle in the cell and shows strong autonomy. To better understand this organelle, we sequenced and analyzed the chloroplast genome of this green alga.

**Principal Findings:**

A total of 111 functional genes, including 69 potential protein-coding genes, 5 ribosomal RNA genes, and 37 tRNA genes were identified. The genome size (153,429 bp), arrangement, and inverted-repeat (IR)-lacking structure of the *B. hypnoides* chloroplast DNA (cpDNA) closely resembles that of *Chlorella vulgaris*. Furthermore, our cytogenomic investigations using pulsed-field gel electrophoresis (PFGE) and southern blotting methods showed that the *B. hypnoides* cpDNA had multimeric forms, including monomer, dimer, trimer, tetramer, and even higher multimers, which is similar to the higher order organization observed previously for higher plant cpDNA. The relative amounts of the four multimeric cpDNA forms were estimated to be about 1, 1/2, 1/4, and 1/8 based on molecular hybridization analysis. Phylogenetic analyses based on a concatenated alignment of chloroplast protein sequences suggested that *B. hypnoides* is sister to all Chlorophyceae and this placement received moderate support.

**Conclusion:**

All of the results suggest that the autonomy of the chloroplasts of *B. hypnoides* has little to do with the size and gene content of the cpDNA, and the IR-lacking structure of the chloroplasts indirectly demonstrated that the multimeric molecules might result from the random cleavage and fusion of replication intermediates instead of recombinational events.

## Introduction

The chloroplast of plants is thought to be descended from an originally free-living cyanobacterium; most of the genes in the genome of the cyanobacterium were transferred to the nucleus of the host cell during the evolutionary transformation of the endosymbiont into the chloroplast [1], [2]. Nevertheless, the chloroplast retained its own genome, which performs some essential metabolic and biosynthetic pathways, such as photosynthesis and amino acid biosynthesis [1].

Chloroplast genomes (cpDNA) were the first plant genomes to be characterized because of their small size, limited number of repeated elements, and abundance of foliar tissues. At present, complete chloroplast genome sequences have been obtained from virtually all of the major higher plant and algal lineages. Comparative analyses of those complete cpDNA sequences not only highlight considerable differences at the organizational level, but also offer clarification of the evolutionary relationships among the main groups of algae and higher plants [3]–[6].

Compared with higher plants, algal chloroplast genomes, especially those of the green algae, exhibit numerous extreme features [7]. The cpDNA of *Helicosporidium* sp. [8], a parasitic, non-photosynthetic green alga, is only 37.5 kb in size, which is the smallest among the cpDNAs characterized, and it lacks all genes for proteins that function in photosynthesis. The cpDNA size of the siphonous alga *Acetabularia* sp. is more than 2000 kb, which is the largest known cpDNA of photosynthetic organisms [1], [9].

The antecedents of higher plants are thought to lie within the green algal lineage [10], [11], so algal plastid genomics offer useful experimental guides for the higher plants. In the past few years, studies of the algal chloroplast genome have been increasing. To date, 14 complete chloroplast genomes have been sequenced for representatives of the chlorophyte lineage: the prasinophytes *Nephroselmis olivacea* [12], *Ostreococcus tauri* [13], *Pyramimonas parkeae* [14], *Pycnococcus provasolii* [14], and *Monomastix sp*. OKE-1 [14]; the trebouxiophytes *Chlorella vulgaris* [15] and *Leptosira terrestris* [16]; the ulvophytes *Pseudendoclonium akinetum* [5], *Oltmannsiellopsis viridis* [17], and *Helicosporidium* sp. [8]; and the chlorophytes *Chlamydomonas reinhardtii* [18] *Scenedesmus obliquus* [19], *Stigeoclonium helveticum* [20], and *Oedogonium cardiacum* [21]. Because the divergence order of these lineages has remained contentious, more sequence data and data from additional taxa are necessary.

In addition to cpDNA sequencing, many studies now are focused on the organization of cpDNA with other elements, such as subgenomic minicircular, plasmid-like molecules [22] and the cpDNA conformation observation [23]–[27]. Using electron microscopy, Kolodner and Tewari [23] found that instead of only monomers, some dimers of cpDNA existed in plant cells. Deng et al. [24] reported that cpDNA can exist in trimer and tetramer form. Using in-gel procedures, including pulsed-field gel electrophoresis (PFGE), restriction fragment mapping, and fluorescence microscopy, Oldenburg and Bendich [28] found that most of the cpDNA from maize seedlings was in linear or complex branched forms rather than in circles. Multimeric forms of cpDNA also have been found in brassicas using field-inversion gel electrophoresis (FIGE) [25] and in tobacco using fluorescence hybridization in situ (FISH) [26]. In contrast, algal cpDNAs, like their higher plant counterparts, have circular restriction maps, and higher order organization (such as multimeric or anomalous forms) has not been identified in algal cpDNAs to date [7].

*Bryopsis* sp., which is a siphonous green alga, is a unicellular coenocytic giant cell. The total contents of the multinucleate cell can be squeezed out, leaving only the cell membranes and walls [29], [30]. The extruded protoplasm without a cell membrane maintains sufficient viability to regenerate into a mature individual [29]. The regenerated individual can grow up to 58 cm, which is three times longer than the wild thalli [30], suggesting that regenerated alga have an advantage over wild individuals in terms of growth. Furthermore, the *Bryopsis* chloroplast is the largest organelle in the cell [31]and is thought to play an important role in the protoplast regeneration process [30], thus we inferred that its cpDNA may be special regarding the genome size and coding gene compared with other algae. However, previous studies of *Bryopsis* almost always have been focused on morphology and mechanisms of regeneration, and the chloroplasts, especially the cpDNAs, have received little attention.

Here we describe the complete sequence of the *B. hypnoides* chloroplast genome and present chloroplast phylogenies based on the genomic data currently available for higher plants and algae. Additionally, we report for the first time the presence of multimeric forms of cpDNA from *B. hypnoides*, a phenomenon that previously was known only for higher plant cpDNAs.

## Results

### Growth of the germinated aggregation of protoplasts of *B. hypnoides*

**Figure 1:**
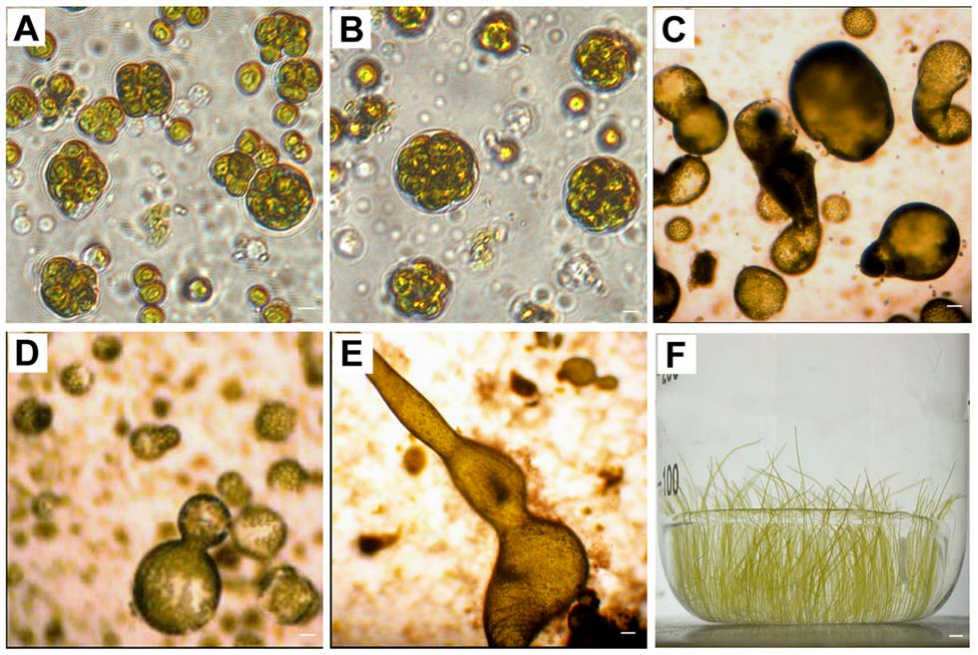
Formation and germination of protoplasts from *B. hypnoides*. A: formation of aggregation of protoplasts within 1 min; B: formation of a gelatinous envelope within 3 min; C–E: germination of the aggregation of *B. hypnoides* protoplasts; F: the germinated aggregation was developed into a mature individual. Bars, 10 µm (A–E), 1cm (F).

The protoplasm extruded from wild *B. hypnoides* immediately aggregated into numerous balls and fine strands when they were mixed with natural seawater (Figure 1A). The aggregations were covered with gelatinous envelopes within 3 min (Figure 1B). Some of the aggregations germinated after 24 hours (Figure 1C–E), and the germinated aggregations then developed into mature individuals (Figure 1F). As in wild *B. hypnoides*, the mature regenerated alga could also develop into the rhizoid, which was used as an anchor, and the thallus.

### Dimensions of chloroplasts in the *B. hypnoides* thallus

**Figure 2:**
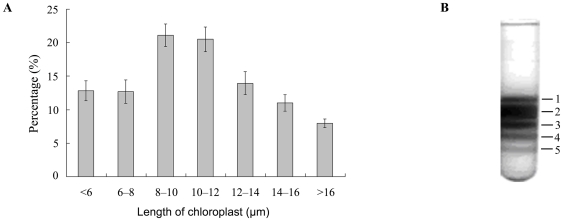
Dimensions of chloroplasts in the *B. hypnoides* thallus. (A) The size distribution of chloroplasts in *Bryopsis hypnoides* thallus as inferred by optical microscope (ZEISS HBO 50, Germany). Ten fields of vision (×200) were selected randomly and all of the chloroplasts with different sizes were determined with an eyepiece micrometer. (B) The chloroplasts separated by sucrose density gradient centrifugation from the wild *B. hypnoides*. Numbers indicate the five discrete bands.

The size of chloroplasts in the *B. hypnoides* thallus varied from 3 to 16 µm, but most were 8–12 µm in length (Figure 2A), suggesting that the size diversity of the chloroplasts existed in the *B. hypnoides* thallus. This result was confirmed by the results of sucrose density gradient centrifugation (Figure 2B), in which five clear discrete green bands appeared in the centrifugation tubes. Then all the bands were recovered separately and observed under the light microscope. We found that all the five bands were intact chloroplasts except for a few cell debris; the chloroplasts in the five bands were of different sizes, demonstrating the presence of five kinds of chloroplasts. When cultured under the same conditions, both the wild and the regenerated algal thallus displayed the same results.

### Structure and gene organization of the *B. hypnoides* chloroplast genome

**Figure 3:**
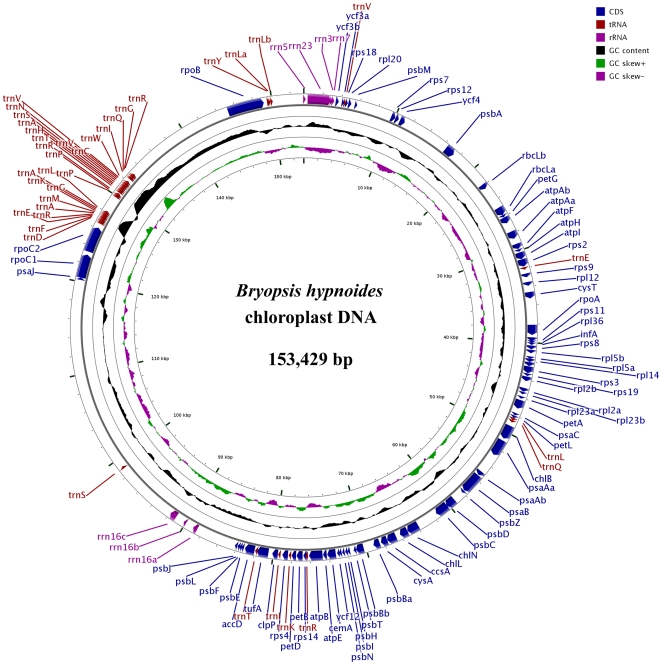
Gene map of the *B. hypnoides* chloroplast genome. Position 0 is in the 12 o'clock position. The CDs of the genes are shown in blue, tRNA genes are indicated as red, and genes of rRNA are shown in violet.

The *B. hypnoides* cpDNA sequence assembles into a circle of 153,429 bp; Figure 3 illustrates its gene map. Overall, the GC content of the cpDNA is 33.1%, which is comparable with that of *Chlamydomonas* (34.5%), *Chlorella* (31.6%), *Pseudendoclonium* (31.5%), and *Pyramimonas* (34.7%). Like its *C. sertularoides* and *C. fragile* homologs, the chloroplast genome of *B. hypnoides* does not contain the inverted repeat that is commonly found in many chloroplast genomes. A total of 111 functional genes, including 69 potential protein-coding genes, 5 ribosomal RNA genes, and 37 tRNA genes, were identified (Table 1). In addition, 29 open reading frames (ORFs) were identified with a threshold of 300 bp. All genes are present in a single copy, and this gene content is typical for chlorophyte cpDNAs. The sequence of *B. hypnoides* was most similar to that of *C. vulgaris* when compared with other completely sequenced chlorophyte cpDNAs. Table 2 compares the gene content of *B. hypnoides* cpDNA with that of other Ulvophyceae, Trebouxiophyceae, and Chlorophyceae (UTC) algal cpDNAs that have been completely sequenced to date. A common set of 84 genes is shared by these genomes. Relative to ulvophytes (*O. viridis* and *P. akinetum*), seven protein genes (*chlI*, *minD*, *psaI*, *psaM*, *rpl19*, *ycf1*, and *ycf20*) are absent from *B. hypnoides* cpDNA. Two genes, *cysA* and *cysT*, that encode sulfate transport proteins are absent in the ulvophytes *O. viridis* and *P. akinetum* but are present in the trebouxiophytes *C. vulgaris* and *L. terrestris* and in our *B. hypnoides* cpDNA.

**Table 1:** Genes contained in *B. hypnoides* chloroplast DNA.

Gene products	Genes
Photosystem I	*psa*A^b^, B, C, J
Photosystem II	*psb*A, B^a^, C, D, E, F, H, I, J, K, L, M, N, T, Z
Cytochrome b6/f	*pet*A, B, D, G, L
ATP synthase	*atp*A^a^, B, E, F, H, I
Chlorophyll biosynthesis	*chl*B, L, N
Rubisco	*rbc*L^b^
Large subunit ribosomal proteins	*rpl*2^b^, 5^b^, 12, 14, 16, 20, 23^b^, 32, 36
Small subunit ribosomal proteins	*rps*2, 3, 4, 7, 8, 9, 11, 12, 14, 18, 19
RNA polymerase	*rpo*A, B, C1, C2
Translation	*inf*A, *tuf*A
Other proteins	accD, *ccs*A, *cem*A, *clp*P, *cys*A, *cys*T
Proteins of unknown function	*ycf*3^b^, 4, 12
Ribosomal RNAs	*rrn*23, 16^aa^, 7, 5, 3
Transfer RNAs	*trnA*(UGC), *A*(CGC), *A*(AGC), *C*(GCA), *D*(GUC), *E*(UUC), *E*(CUC), *F*(GAA), *G*(UCC), *G*(GCC), *H*(GUG), *I*(AAU), *I*(GAU), *K*(CUU), *K*(UUU), *L*(CAA), *L*(UAA)^a^, *L*(UAG), *M*(CAU), *N*(GUU), *P*(UGG), *P*(AGG), *Q*(CUG), *Q*(UUG), *R*(ACG), *R*(CCU), *R*(UCG), *R*(UCU), *S*(GCU), *S*(UGA), *T*(AGU), *T*(UGU), *V*(AAC), *V*(CAC), *V*(UAC), *W*(CCA), *Y*(GUA)

a Genes containing one- and two- group I introns.

b Genes containing group II introns.

**Table 2:** Gene Content in *B. hypnoides* and Other UTC Algal cpDNAs.

Gene	Cv	Lt	Ov	Pa	By	Cr	So	Sh	Oc
*accD*	•	•	•	•	•	○	○	○	○
*ccsA*	•	○	•	•	•	•	•	•	•
*chlB*	•	•	•	○	•	•	•	•	•
*chlI*	•	○	•	•	○	○	○	○	○
*chlL*	•	•	•	○	•	•	•	•	•
*chlN*	•	•	•	○	•	•	•	•	•
*cysA*	•	•	○	○	•	○	○	○	○
*cysT*	•	•	○	○	•	○	○	○	○
*ftsH*	○	•	○	○	○	○	○	•	•
*infA*	•	•	•	•	•	○	•	○	•
*minD*	•	•	•	•	○	○	○	○	○
*petA*	•	•	•	•	•	•	•	○	○
*psaI*	•	•	•	•	○	○	○	○	○
*psaM*	•	•	•	•	○	○	○	•	•
*rpl12*	•	•	•	•	•	○	•	○	○
*rpl19*	•	•	•	•	○	○	○	○	○
*rpl32*	•	•	•	•	•	○	○	•	•
*rpoC1*	•	•	•	•	•	○	•	•	•
*rrn3*	○	○	○	○	•	•	○	○	○
*rrn7*	○	○	○	○	•	•	○	○	○
*ycf1*	•	•	•	•	○	○	•	•	•
*ycf12*	•	○	•	•	•	•	•	•	•
*ycf20*	•	•	•	•	○	○	○	○	○
*ycf62*	•	•	○	•	○	○	○	○	○
*trnA*(cgc)	○	○	○	○	•	○	○	○	○
*trnA*(agc)	○	○	○	○	•	○	○	○	○
*trnE*(cuc)	○	○	○	○	•	○	○	○	○
*trnL*(caa)	•	•	○	•	•	○	○	•	•
*trnL*(gag)	•	○	○	○	○	○	○	○	○
*trnI*(aau)	○	○	○	○	•	○	○	○	○
*trnI*(cau)	•	•	•	•	○	•	•	•	•
*trnK*(cuu)	○	○	○	○	•	○	○	○	○
*trnP*(agg)	○	○	○	○	•	○	○	○	○
*trnQ*(cug)	○	○	○	○	•	○	○	○	○
*trnR*(ccg)	•	•	○	•	○	○	○	○	○
*trnR*(ccu)	○	○	•	•	•	○	○	○	•
*trnR*(ucg)	○	○	○	○	•	○	○	○	•
*trnS*(gga)	•	○	○	○	○	○	○	•	○
*trnT*(agu)	○	○	○	○	•	○	○	○	○
*trnT*(ggu)	•	○	○	○	○	○	○	○	○
*trnV*(cac)	○	○	○	○	•	○	○	○	○
*trnV*(aac)	○	○	○	○	•	○	○	○	○

Cv: *Chlorella vulgaris*, Lt: *Leptosira terrestris*, Ov: *Oltmannsiellopsis viridis*, Pa: *Pseudendoclonium akinetum*, By: *Bryopsis hypnoide*, Cr: *Chlamydomonas reinhardtii*, So: *Scenedesmus obliquus*, Sh: *Stigeoclonium helveticum*, Oc: *Oedogonium cardiacum*. A filled/open circle denotes the presence/absence of a gene. Only the genes that are missing in one or more genomes are indicated. A total of 84 genes are shared by all compared cpDNAs: *atpA, B, E, F, H, I, cemA, clpP, petB, D, G, L, psaA, B, C, J, psbA, B, C, D, E, F, H, I, J, K, L, M, N, T, Z, rbcL, rpl2, 5, 14, 16, 20, 23, 36, rpoA, B, C2, rps2, 3, 4, 7, 8, 9, 11, 12, 14, 18, 19, rrf, rrl, rrs, tufA, ycf3, 4, trnA*(ugc), *C*(gca), *D*(guc), *E*(uuc), *F*(gaa), *G*(gcc), *G*(ucc), *H*(gug), *I*(gau), *K*(uuu), *L*(uaa), *L*(uag), *Me*(cau), *Mf*(cau), *N*(guu), *P*(ugg), *Q*(uug), *R*(acg), *R*(ucu), *S*(gcu), *S*(uga), *T*(ugu), *V*(uac), *W*(cca), *Y*(gua).

In terms of gene organization, many derived gene clusters are shared specifically between *B. hypnoides, C. vulgaris, O. viridis*, and *P. akinetum* cpDNAs (i.e., *rpl16-rpl14-rpl5-rps8-infA-rpl36-rps11-rpoA, rps2-atpI-atpH-atpF-atpA, psbE-psbF-psbL-psbJ*, and *ccsA-chlL-chlN* (not in *P. akinetum*)), as are gene pairs (i.e., *atpB-atpE, petB-petD, rpl2-rps19, rps12-rps7, psbD-psbC, rpoC1-rpoC2, rps19-rps3, rps9-rpl12, rps2-atpI, rpl20-rps18, psbK-ycf12*, and *psaA-psaB* (not in *P. akinetum*)) as well as two ancestral gene pairs (*psbB-psbT* and *rpl23-rpl2*). The gene pairs *rps*3-*rpl*16, *rpoB-rpoC1*, and *tufA-rpl19* and the *petA-petL-petG* cluster are missing from our *B. hypnoides* cpDNA.

Eleven introns in the *B. hypnoides* chloroplast are distributed among ten genes, among which *rrs* exhibits two introns and *atpA*, *psaA*, *psbB*, *rbcL*, *rpl2*, *rpl5*, *rpl23*, *trnL*-UAA and *ycf3* each contain one intron. These introns vary from 348 to 2466 bp in size. According to their secondary structures, five introns belong to the group I family [32], and three of these carry an internal ORF encoding a putative LAGLIDADG homing endonuclease. The intron of *rpl2* is commonly present in the chloroplast genomes of land plants, however it not found in the completely sequenced chlorophyte cpDNAs. The introns of *rpl5* and *rpl23* in *Bryopsis* are the first found in the known chloroplast genomes of Viridiplantae, and blast searches of these two intron sequences against the GenBank database failed to detect any homologous introns in other organisms.

### PFGE analysis of cpDNA

**Figure 4:**
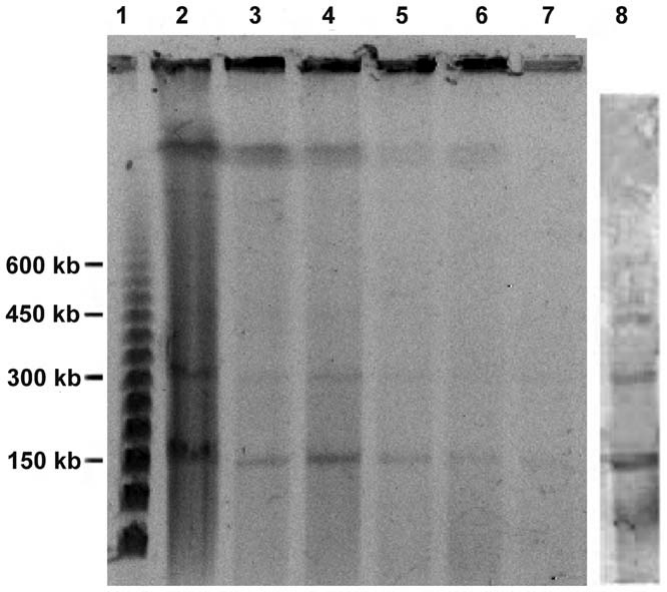
PFGE showing multimeric forms of chloroplasts from the wild (A) and the regenerated (B) *B. hypnoides*. (A) lane 1: molecular standard marker; lane 2: cpDNAs from crude chloroplasts of *B. hypnoides* without purification by sucrose density gradient centrifugation; lanes 3–7: cpDNAs from different chloroplasts separated by sucrose density gradient centrifugation; lane 8: the cpDNA hybridized with the labeled probe of *rbcL* gene. (B) lane 1: molecular standard marker; lanes 2–6: cpDNAs from different chloroplasts separated by sucrose density gradient centrifugation (corresponding to the bands 1–5 in Fig. 3); lane 7: the cpDNA hybridized with the labeled probe of *rbcL* gene.

**Figure 5:**
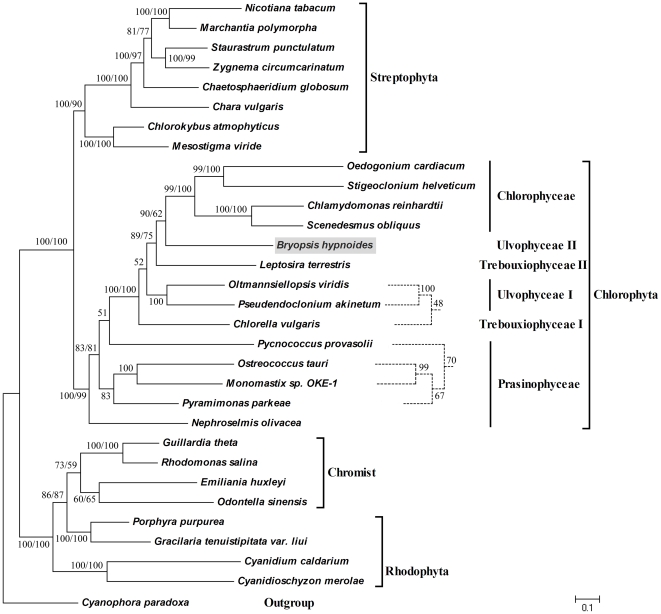
Phylogenetic position of *B. hypnoides* as inferred by ML analyses of 42 cpDNA-encoded proteins. Family-level affinities are shown on the right of the diagram. *Cyanophora paradoxa* were used as outgroup. Numbers in each branch indicated maximum-likelihood (ML) / maximum parsimony (MP) bootstrap values; and the dashed lines indicated the MP topologies which were different from the ML tree.

Figure 4A shows the results of PFGE analysis of cpDNAs of the wild *B. hypnoides*. In the cpDNAs from the different types of chloroplasts purified by sucrose density gradient centrifugation, at least four clear bands can be seen in every lane, each corresponding to a type of *B. hypnoides* chloroplast. The southern hybridization with labeled probes of the *rbcL* gene showed all the four bands were positive (Figure 5), suggesting that the four bands represented all of the cpDNAs from *B. hypnoides*. The four bands were located at 150 kb, 300 kb, 450 kb, and 600 kb, all of which were in multiple relations. The relative amounts of the four bands in PFGE, which corresponded to monomers, dimers, trimers, and tetramers of the *B. hypnoides* cpDNAs, were estimated to be about 1, 1/2, 1/4, and 1/8 based on molecular hybridization.

Figure 4B shows the results of PFGE analysis of cpDNAs from the regenerated *B. hypnoides*. These results are almost the same as those shown in Figure 4A, which confirms that the cpDNAs from the regenerated individual are similar to those of the wild alga.

### Phylogenetic position of *B. hypnoides* chloroplasts

To elucidate the overall position of *B. hypnoides* in the plastid phylogeny of algae/land plants, a global analysis was performed using a subset of 31 taxa (see supplemental data) and 14,160 aligned characters. Thirty-one organisms were included as representatives of algae and higher plants and included the green lineage (streptophyte and chlorophyte lineage) and the non-green lineage (red and chromist lineage). The best tree identified two distinct lineages: the green lineage and the non-green lineage. Moreover, the chlorophytes and streptophytes formed two distinct green lineages (Figure 5). Unexpectedly, the trees inferred with ML and MP methods both identified a clade uniting *B. hypnoides* and four complete sequenced members of the Chlorophyceae, this clade received 90% and 62% bootstrap value in MP and ML analyses, respectively. For other chlorophytes, *N. olivacea* represents the first branch of the Chlorophyta, which is also reported in recent phylogenetic analyses [6], [12], but the branching order of the other four Prasinophyceae (*O. tauri*, *P. parkeae*, *P. provasolii* and *Monomastix sp*.) showed the different topology in the ML and MP analyses. In ML tree, *P. provasolii* is sister to all other UTC algae (bootstrap 51%); in the MP topology, it forms a moderately supported clade with *O. tauri*, *Monomastix sp*. and *P. parkeae*. For the UTC algae, the topological differences are also seen in the *C. vulgaris* clade, which was clustered with two Ulvophyceae (*O. viridis* and *P. akinetum*) in the MP tree, whereas in the ML tree, it located at the basal position within the UTC algae.

The relationships observed for the non-green algae and streptophytes taxa in the phylogeny are congruent with recently published phylogenies based on whole chloroplast genome sequences [6], [33]. Within the Streptophyta, the clade uniting *Chlorokybus* and *Mesostigma* was placed basally and received strong bootstrap support. The other four Charophyceae were clustered with two higher plants (*N. tabacum* and *M. polymorpha*). In terms of the non-green lineage examined in this study, *P. purpurea* and *G. tenuistipitate var. liui* formed a strongly supported lineage that is sister to the clade uniting the *G. theta*, *R. salina*, *O. sinensis* and *E. huxleyi*. Two other red algae, *C. merolae* and *C. caldarium*, robustly clustered in a separate clade.

## Discussion

### The autonomy of the *B. hypnoides* chloroplast

Our previous studies showed that the chloroplasts of *B. hypnoides* had strong autonomy for the following reasons: (1) The chloroplasts in *B. hypnoides* had greater vitality than other organelles under unfavorable conditions [34]; (2) chloroplasts purified by sucrose density gradient centrifugation can aggregate into spheres, although they cannot develop into mature individuals [35]; and (3) The chloroplasts from siphonous algae possess great vitality and can maintain activity in other cells or other body cavities. For example, a symbiotic association exists between siphonaceous green algae (*Acetabularia, Bryopsis*, *Caulerpa*, and *Codium*) plastids and some marine sea slug species that are highly specialized herbivores that feed on siphonalean algae by puncturing the cells and sucking out the contents. The chloroplasts of siphonous algae are not always digested and can lodge in the body of the animal and conduct photosynthesis for at least 3 months [36]–[41]. Moreover, genes supporting photosynthesis have been acquired by the host animal via horizontal gene transfer, and the encoded proteins are retargeted to the chloroplast [42]. In summary, compared with chloroplasts of other algae and higher plants, *B. hypnoides* chloroplasts have great vitality and independence. Because of these extraordinary traits, we conducted research both on the genome sequence and the conformation of the cpDNA.

### Distinctive features of the *B. hypnoides* chloroplast genome

The genome size, arrangement, and IR-lacking structure of the chloroplast genome of *B. hypnoides* more closely resemble that of *C. vulgaris* cpDNA [15] than its *O. viridis* [17] and *P. akinetum* [5] homologs. In addition to the 84 conserved genes that exist in all of the completely sequenced UTC algal cpDNAs, *B. hypnoides* shares an additional 14 genes with *C. vulgaris* cpDNA, compared to 12 and 10 genes with *O. viridis* and *P. akinetum*, respectively (Table 2).

Among the chlorophycean algal plastids investigated to date, the inverted repeat is lost in the prasinophytes (*Monomastix* and *Pycnococcus* [14]); trebouxiophytes (*Chlorella* [15], *Helicosporidium* [8], and *Leptosira* [16]); ulvophytes (*Caulerpa* [43] and *Codium* [44]); and the chlorophyte *Stigeoclonium* [20], suggesting that this ancestral character has been independently lost in those lineages. Furthermore, considering the atypical quadripartite structure of *Oltmannsiellopsis* and *Pseudendoclonium* cpDNAs identified previously, the chloroplast genome of the Ulvophyceae likely has evolved under relaxed constraints [5].

The most notable feature of the *B. hypnoides* chloroplast genome is that the rRNA locus consists of five genes: *rrn23*, *rrn16*, *rrn7*, *rrn5*, and *rrn3*. The same situation can be found only in *C. reinhardtii* cpDNA [18], as genes *rrn7* and *rrn3* are absent from all other completely sequenced chlorophyte cpDNAs. Similar to *C. reinhardti*, the rRNA gene cluster of *B. hypnoides* is arranged in the order *rrn16*- *rrn5*- *rrn23*- *rrn3*-*rrn7*; however, the typical rRNA operon in *B. hypnoides* has been broken in half and the SSU and LSU genes are distributed at opposite ends of the gene map circle, as is found in the ulvophytes *C. sertularoides* [43] and *C. fragile* [44]; this might be an outcome of the loss of the inverted repeat.

Another surprising feature of the *B. hypnoides* chloroplast gene repertoire is the presence of 10 unusual tRNA genes that have not been found in other completely sequenced chlorophyte cpDNAs. Five of them (*trnA*-AGC, *trnE*-CUC, *trnI*-AAU, *trnV*-CAC, and *trnV*-AAC) correspond to those identified in some embryophyte cpDNAs, whereas the other five (*trnA*-CGC, *trnK*-CUU, *trnP*-AGG, *trnQ*-CUG, and *trnT*-AGU) have been found previously only in some bacterial genomes. These unusual tRNA genes may not be essential for plastid function in green algae or may not be functional genes; they also might be involved in the special physiological functions of *B. hypnoides*.

Wolfe et al. [45] reported that the cp genome of *Epifagus virginiana,* a plant with non-photosynthetic chloroplasts, encodes only 25 proteins because the photosynthetic machinery and the corresponding genes are not needed. Glöckner et al. [46] identified several genes unique to the cp genome of *C. caldarium* that correlate to its special environmental conditions. Thus, it seems that loss or gain of function is accompanied by changes of genes in the cp genome. Herein we originally presumed that the size and gene content of the chloroplast genome of *B. hypnoides* should be extraordinary in order to support its autonomy. However, we found that *B. hypnoides* possesses the usual chloroplast genome of 153 kbp and that the gene repertoire is typical for chlorophyte cpDNAs. Thus, the autonomy of chloroplasts of *B. hypnoides* seems to have little to do with the size and gene content of the cpDNA.

### The multimeric forms conformation of *B. hypnoides* cpDNA

Figure 4 shows that the cpDNA of *B. hypnoides* has multimeric forms, including at least monomers, dimers, trimers, and tetramers, and these are similar to the multimeric cpDNA forms of higher plants, such as *Arabidopsis*, tobacco, and peas [24], [26], [47]. The five different sizes of *B. hypnoides* chloroplasts showed the same cpDNA characteristics in multimeric forms, suggesting that the relative amounts of the different forms of *B. hypnoides* cpDNA had no relationship to chloroplast dimension.

Green alga is thought to have been the progenitor of higher plants [10]–[12]; *B. hypnoides* is a green alga thus, its cpDNA conformation may be similar to that of higher plants. However, a PFGE study of the *C. reinhardtii* chloroplast genome did not reveal the higher order organization that was observed previously for higher plant cpDNAs [18]. Our phylogenetic tree revealed that *B. hypnoides* is located in the same clade as *C. reinhardtii* (Figure 5), whereas their cpDNA conformations were quite different. This finding indicates that the cpDNA of green algae is structurally plastic.

Lilly et al. [26] proposed that the formation of higher order multimeric molecules may result from recombinational events or the random cleavage and fusion of replication intermediates. Recombinational events correlate to the presence of IRs, as revealed in both *Arabidopsis* and tobacco [26], [48]. Recombination between IRs that occurs on two separate monomers results in a dimer [49]; subsequent recombination events between molecules presumably produce multimers. However, our study demonstrated that multimeric forms of cpDNA also exist in the IR-lacking chloroplast genome, which suggests that the higher order organization of the chloroplast genome maybe less related to the presence of IRs than was previously thought. Our results indirectly support the alternative explanation that multimeric molecules were produced by the random cleavage and fusion of replication intermediates. However, this explanation requires confirmation further.

### Similar conformation of cpDNA between wild and regenerated *B. hypnoides*

Our previous studies showed that the regenerated alga had an advantage over the wild individual in terms of growth and that biochemical compositions differed between the wild and the regenerated alga [30], [34]. The regenerated alga can grow up to 58 cm in length, which is three times longer than the wild form [30]. Wang and Tseng [34] reported that when the regenerated alga was on the decline, the organelles aggregated in the thallus and then moved to the outside; next, one organelle aggregation located outside of the thallus germinated and developed into a mature alga. From this, we inferred that the DNA in the organelles of the regenerated alga, especially the cpDNAs, underwent changes during organelle aggregation and later development into a mature individual. The cpDNAs from the regenerated *B. hypnoides* were similar in size and conformation to the cpDNA of the wild alga (Figure 4), and both exhibited monomeric, dimeric, trimeric, and tetrameric forms of cpDNAs. Thus, the differences between the wild alga and the regenerated individual probably result from gene expression rather than alteration of the genome, especially the cpDNAs.

### Evolution of the chlorophycean chloroplast genome

The basal position of the Prasinophyceae in the Chlorophyta is well established, but the branching order of the Ulvophyceae, Trebouxiophyceae, and Chlorophyceae remains unresolved [5], [51]. There are two hypotheses concerning the divergence order of the UTC lineages: 1) phylogenetic inferences from cpDNA-encoded proteins and genes favor the hypothesis that the Ulvophyceae is a sister to the Trebouxiophyceae [5]; and 2) chloroplast phylogenies inferred from gene order [5] and mitochondrial phylogenies inferred from proteins or genes [51] suggest that the Ulvophyceae share a sister relationship with the Chlorophyceae. Our phylogenetic analyses of 42 chloroplast proteins revealed that the Ulvophyceae clade I (*O. viridis* and *P. akinetum*) is a sister to the Trebouxiophyceae I (*C. vulgaris*). However, the *B. hypnoides* (Ulvophyceae II) chloroplast genome is closely related to the Chlorophyceae. Although the *B. hypnoides* chloroplast genome shares similarities with its *O. viridis* and *P. akinetum* counterparts in terms of gene order, its IR-lacking structure and gene content are quite different; we therefore inferred that Ulvophyceae was non-monophyletic group, *B. hypnoides* is located in the different phylogenetic lineage with *O. viridis* and *P. akinetum*. Moreover, our phylogenetic analyses favor the premise that the Trebouxiophyceae and Prasinophyceae were also non-monophyletic groups, thus it was difficult to state a precise taxonomic relationship among the UTC lineages in this study. These different phylogenetic results may be caused by the insufficient taxon sampling. So studies on additional chloroplast genome data, especially from the UTC algae, will be very useful for determining the phylogenetic relationships among the major lineages of Chlorophyta.

## Materials and Methods

### Materials

*Bryopsis hypnoides* Lamouroux was collected from the intertidal zone of Zhanqiao Pier, Qingdao, China (36°3′N, 120°19′E). The fresh algae, which were rinsed with plenty of autoclaved seawater and brushed with a soft brush to remove the surface microbial and epiphytic organisms, were cultured in autoclaved sea water under irradiance of 25 µmol m^−2^ · s^−1^ with a 16 h light: 8 h dark regime at room temperature [30].

### The aggregation of organelles in the protoplasm

Thalli of *B. hypnoides* were cut into small pieces and then placed in eight layers of sterilized gauze to squeeze out the protoplasm. The extruded protoplasm was mixed with an equal volume of sterilized seawater (pH 8.3) and gently rocked. The organelles in the protoplasm aggregated into spheres of different sizes, and the aggregated spheres were cultured into mature *B. hypnoides* (the regenerated *B. hypnoides*) under the culture conditions described above.

### Chloroplast and cpDNA purification

The protoplasts squeezed from the wild *B. hypnoides* were added to double volume ice-cold extraction buffer (400 mM sucrose, 50 mM Tris, 20 mM EDTA, 0.2% BSA, 0.2% β-mercaptoethanol, pH 7.8) and then filtered through four layers of cheese cloth. The filtrate was centrifuged at 800 g at 4°C for 10 min, and the pellet was suspended in the extracted buffer. Most of the pellets were found to be chloroplasts under microscopic examination, so they were layered onto the sucrose density gradient from 10% to 60%. The gradient was centrifuged at 150,000g for 90 min, and five clear green bands appeared in the tube after centrifugation. The bands were removed from the tube separately, and dialyzed against the rinse buffer (400 mM sucrose, 50 mM Tris, 0.5%, BSA pH 7.8) for 5 hours to remove sucrose, then were observed under the microscope.

The purified chloroplasts were washed twice with the buffer (50 mM Tris-HCl, pH 8.0, 25 mM EDTA), suspended in the lysis buffer (50 mM Tris, pH 8.0, 25 mM EDTA, 2% SDS, 50 µg/ml proteinase K), and incubated at 40°C for 3 hours with gently shaking, following by being centrifuged at 10 000 g at 4°C for 15 min. The supernatant was extracted several times with phenol/chloroform and then precipitated with cold ethanol. CsCl density gradient ultracentrifugation was used for further purification of the cpDNA. CsCl and Hoechst dye No. 33258 were added to the crude cpDNA, and the mixture was centrifuged at 240,000g at 20°C for 38 hours with a Beckman Ti 80 rotor. cpDNA bands were visualized under UV illumination and then recovered. The cpDNA was precipitated and dissolved in TE buffer after both the Hoechst dye 33258 and CsCl were removed. The cpDNA from the regenerated *B. hypnoides* then was purified as described above.

### cpDNA sequencing

The purified cpDNA was sheared by nebulization, and 1,500±3,000 bp fragments were recovered by electroelution after agarose gel electrophoresis. These fragments were treated with T4 DNA polymerase and cloned into the SmaI site of PUC18. After transformation of electrocompetent *E. coli* TOP10 cells (Invitrogen, Carlsbad, CA), recombinant plasmids were isolated and nucleotide sequences were determined with the PRISM dye terminator cycle sequencing kit (Applied Biosystems, Foster City, CA) on a DNA sequencer (model 373; Applied Biosystems) using T3 and T7 primers. Sequencing data were accumulated to 10× coverage for all PCR fragments; remaining gaps were cloned by PCR. The determined sequences were accumulated, trimmed, aligned, and assembled using the Phred-Phrap (Phil Green, University of Washington, Seattle, WA, USA) and Consed programs [52]. The fully annotated *B. hypnoides* chloroplast genome sequence has been deposited in GenBank with accession number GQ892829.

### PFGE and southern blot analysis

The PFGE assay was performed by the method described by Sambrook and Russell [53]. Brifely, the purified chloroplasts were mixed with an equal volume of low melting point agarose, which was dissolved in rinse buffer containing 20 mM EDTA, and 90 µl of the mixture was added into every pole of the mould (Bio-Rad, Richmond, Calif), then solidified at 4°C. The solid gels were put into the lysis buffer (0.01 M Tris, 0.45 M EDTA, pH 7.8, 2% SLS, 10 µg/ml proteinase K) and incubated at 50°C for 40 h, during which the buffer was changed twice. The gels then were rinsed six times with TE (10 mM Tris, 1 mM EDTA, pH 8.0) and then stored at 4°C. Pulsed-field gel electrophoresis (PFGE) was performed on a Bio-Rad CHEF Mapper TM according to the manufacturer's instructions (CHEF Mapper TM and CHEF Mapper XA Pulsed Field Electrophoresis Systems). After PFGE, the gel was dyed with 0.5 µg/ml EB for 30 min, and the result was observed by Pharmacia Biotech Imagemaster® VDS.

A DNA fragment encoding the *B. hypnoides rbcL* gene (1145 bp; GenBank accession no. AY566304) was prepared from genome DNA as described previously [54]. The *rbcL* gene then was labeled according to the directions provided in the Dig High Prime DNA Labeling and Detection Starter Kit I (Roche, Germany) and used as a probe for southern blot analysis. DNA in the gel was denatured and transferred to the positively charged nylon membrane (Osmonics, Westborough, MA, USA) [53] and cross-linked under UV for 90 seconds, then hybridized with the probe following the instructions for the Roche Kit.

### Sequence and phylogenetic analyses of cpDNA

Genes were identified using Blast homology searches provided by the National Center for Biotechnology Information (NCBI) server (http://www.ncbi.nlm.nih.gov/BLAST/). Protein-coding genes and positions of ORFs were determined using ORFFINDER at NCBI. tRNA genes were annotated using the tRNAscan-SE program [57]. Intron boundaries were located by modeling intron secondary structures [32], [56] and by comparing the sequences of intron-containing genes with those of intron-less homologues using FRAMEALIGN of the Wisconsin package. Circle graphs were generated using the CGView program [57].

Phylogenetic analysis was conducted using forty-two cp protein sequences (*atpA*, *atpB*, *atpE*, *atpF*, *atpH*, *petB*, *petD*, *petG*, *psaA*, *psaB*, *psaC*, *psaJ*, *psbA*, *psbB*, *psbC*, *psbD*, *psbE*, *psbF*, *psbH*, *psbI*, *psbJ*, *psbK*, *psbN*, *psbT*, *psbZ*, *rpl2*, *rpl14*, *rpl16*, *rpl20*, *rpl36*, *rps2*, *rps3*, *rps4*, *rps7*, *rps8*, *rps11*, *rps12*, *rps14*, *rps18*, *rps19*, *ycf3*, and *ycf4*) from 31 algal/land plant organisms (see Data S1). The concatenated protein sequences were aligned using the multiple sequence alignment tools in CLUSTAL X version 1.81 with the default settings [58]. The adjusted alignment after manual correction was used for phylogenetic analyses by maximum likelihood (ML) and maximum parsimony (MP) methods. ML trees were computed with PHYML 3.0 [59] under the cpREV45+Γ+I model of amino acid substitutions [60] and bootstrap support for each node was calculated using 100 replicates. MP trees were calculated with MEGA 4.0 [61] by 1,000 bootstrap replications, which were obtained using the Close-Neighbor-Interchange algorithm. MEGA 4.0 was used for visualization and printing of the trees.

## Supporting Information

Data S1 Algal and land plant chloroplast genomes examined in the phylogenetic analyses
(0.02 MB DOC)
