# Correction: The *Bryopsis hypnoides* Plastid Genome: Multimeric Forms and Complete Nucleotide Sequence

**DOI:** 10.1371/annotation/688b429f-9a1e-4ca8-8489-52f5609f26a4

**Published:** 2012-03-27

**Authors:** Fang Lü, Wei Xü, Chao Tian, Guangce Wang, Jiangfeng Niu, Guanghua Pan, Songnian Hu

There was an error in the second affiliation. The correct second affiliation is 2 CAS Key Laboratory of Genome Sciences and Information, Beijing Institute of Genomics, Chinese Academy of Sciences(BIGCAS), Beijing, China. 

