# Supplementary material for: The Bryopsis hypnoides Plastid Genome: Multimeric Forms and Complete Nucleotide Sequence
Source: PLoS One. 2011 Feb 14;6(2):e14663. doi: 10.1371/journal.pone.0014663 (PMC3038852; doi:10.1371/journal.pone.0014663)
Supplement: Data S1 — Algal and land plant chloroplast genomes examined in the phylogenetic analyses (0.02 MB DOC) [file pone.0014663.s001.doc]

*Chaetosphaeridium* *globosum* [AF494278], *Chara vulgaris* [DQ229107], *Chlamydomonas* *reinhardtii* [BK000554], *Chlorella vulgaris* [AB001684], *Chlorokybus atmophyticus* [DQ422812], *Cyanidium caldarium* [NC_001840], *Cyanidioschyzon merolae* [NC_001799], *Cyanophora paradoxa* [U30821], *Gracilaria tenuistipitata var. liui* [NC_006137], *Guillardia theta* [NC_000926], *Leptosira terrestris* [EF506945], *Marchantia polymorpha* [NC_001319], *Mesostigma viride* [AF166114], *Monomastix sp. OKE-1* [NC_012101], *Nephroselmis olivacea* [AF137379], *Nicotiana tabacum* [NC_001879], *Odontella sinensis* [NC_001713], *Oedogonium cardiacum* [EU677193], *Oltmannsiellopsis viridis* [DQ291132], *Ostreococcus tauri* [CR954199], *Porphyra purpurea* [NC_000925], *Prochlorococcus marinus str. MIT 9313* [NC_005071], *Pseudendoclonium akinetum* [AY835431], *Pyramimonas parkeae* [NC_012099], *Pycnococcus provasolii* [NC_012097], *Rhodomonas salina* [NC_009573], *Scenedesmus obliquus* [DQ396875], *Spinacia oleracea* [NC_002202], *Staurastrum punctulatum* [NC_008116], *Stigeoclonium helveticum* [DQ630521], *Zygnema circumcarinatum* [NC_008117].
